# The association between childhood adversity and hippocampal volumes is moderated by romantic relationship experiences

**DOI:** 10.1111/ejn.16593

**Published:** 2024-11-17

**Authors:** H. Acosta, A. Jansen, T. Kircher

**Affiliations:** ^1^ Department of Psychiatry and Psychotherapy Philipps University Marburg Germany; ^2^ The FinnBrain Birth Cohort Study, Turku Brain and Mind Center, Department of Clinical Medicine University of Turku Finland; ^3^ Core Facility Brainimaging, Faculty of Medicine Philipps University Marburg Germany

**Keywords:** attachment, childhood maltreatment, early life stress, loss, MRI

## Abstract

Reduced hippocampal volumes are a feature of many mental disorders. Childhood maltreatment is a known risk factor for the development of psychopathology and has consistently been linked to hippocampal volume reductions in adults, but not in children and adolescents. We propose that maltreatment‐related difficulties in coping with developmental tasks in adolescence and young adulthood might underlie the delayed emergence of hippocampal volume reductions in maltreated individuals. In a study with 196 healthy young adults (mean age [years]: 24.0 ± 3.2, 50% female, 20.6% living with a partner (missings: n = 2)), we investigated the interaction between childhood maltreatment (Childhood Trauma Screener) and the breakup of a steady romantic relationship (List of Threatening Experiences Questionnaire) on hippocampal magnetic resonance imaging grey matter volumes. The experience of a romantic relationship breakup moderated the association between childhood maltreatment and bilateral hippocampal volumes, revealing more negative associations with hippocampal volumes in participants with at least one breakup compared to those with no breakup experience (right hippocampus: β = − 0.05 ± 0.02, p = 0.031, p (FDR) = 0.031; left hippocampus: β = −0.06 ± 0.02, p = 0.005, p (FDR) = 0.009). Moreover, our findings provide some evidence that childhood maltreatment is related to smaller bilateral hippocampal volumes only in those adults who suffered from a relationship breakup (right hippocampus: β = −0.23 ± 0.10, p = 0.018, p (FDR) = 0.018; left hippocampus: β = −0.24 ± 0.10, p = 0.016, p (FDR) = 0.018;). Our study highlights the interaction of adult social bonds with early adversity on vulnerability to psychopathology.

## Graphical abstract

We investigated in 196 young healthy adults how childhood maltreatment interacts with romantic relationship breakup experiences on hippocampal grey matter volumes. Significantly smaller bilateral hippocampal volumes were associated with more severe childhood maltreatment (CTS Sum) in persons with a romantic relationship breakup experience compared to those with none. Our results support a vulnerability‐stress‐interaction for hippocampal volumes in young healthy adults.

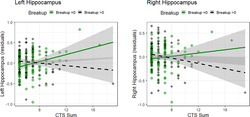

**Abbreviations**
ANX: Attachment anxiety
AV: Attachment avoidance
BDI: Beck Depression Inventory
*breakup*
: Romantic relationship breakup
CTS: Childhood Trauma Screener
ES: Adjusted effect size
FDR: False‐discovery‐rate
LTE‐Q: List of Threatening Experiences Questionnaire
MDD: Major depressive disorder
PTSD: Posttraumatic stress disorder
RSQ: Relationship Scales Questionnaire
SD: Standard deviation
SE: Standard error
STAI‐T: State–Trait Anxiety Inventory, Trait version
TAS: Toronto Alexithymia Scale
TIV: Total intracranial volume
vs: versus

## 1 INTRODUCTION

Childhood maltreatment is a risk factor for most adult psychiatric disorders such as major depressive disorder (MDD), schizophrenia, posttraumatic stress disorder (PTSD) and personality disorders (Bailey et al., 2018; Kessler et al., 2010; McGinnis et al., 2022; McLaughlin et al., 2017; Reuben et al., 2016; Sahle et al., 2022; Tan & Mao, 2023; Varese et al., 2012). Typically, a dose–response relationship exists between the severity of childhood maltreatment and the likelihood of psychiatric disorders (Hughes et al., 2017; Tan & Mao, 2023). It has been hypothesized that childhood maltreatment renders the individual more vulnerable to subsequent stressful life events by shaping the individual's neurodevelopment (Ringwald, Pfarr, Schmitt, et al., 2022; Ringwald, Pfarr, Stein, et al., 2022), partly in interaction with genetic variations in neurotransmitter transporters, neurotrophic factors (Heim et al., 2008) and hippocampal glucocorticoid receptors (Buchmann et al., 2014).

From a neurodevelopmental perspective, the hippocampus has gained attention as a potential connecting structure between childhood maltreatment and adult psychiatric disorders (Teicher et al., 2012; Teicher & Samson, 2016). Reduced hippocampal volumes, predominantly of the left hemisphere, are a common feature in several psychiatric disorders such as MDD, PTSD, bipolar disorder and schizophrenia (Bromis et al., 2018; Brosch et al., 2022; Espinoza Oyarce et al., 2020; Goodkind et al., 2015; Roeske et al., 2021; Santos et al., 2018; Sun et al., 2023). Hippocampal volumes were smaller in adults with a history of childhood maltreatment (Calem et al., 2017; Paquola et al., 2016), independent of disease status (Hakamata et al., 2022; Opel et al., 2014). Hippocampal volume reductions were also correlated with the severity of childhood maltreatment (Riem et al., 2015).

The question remains why childhood maltreatment is fairly consistently associated with reduced hippocampal volumes in adulthood, but not in childhood or adolescence (Hakamata et al., 2022; Teicher & Samson, 2016): Findings are mixed among child and adolescent studies. For example, in human studies of children and adolescents, not only reduced (Dahmen et al., 2018; Edmiston et al., 2011; Lee et al., 2018; Malhi et al., 2019), but also unaltered (Carrion et al., 2001; De Bellis et al., 2001; Malhi et al., 2019; Whittle et al., 2017) or even increased (Tupler & De Bellis, 2006; Whittle et al., 2013) hippocampal volumes have been shown. A seminal translational study exposed rats to early traumatic stress (Andersen & Teicher, 2004). The results indicated that hippocampal volume reductions did not emerge before adulthood. No comparable volume trajectories were observed in the rat amygdala or prefrontal cortex, highlighting brain regional differences (Andersen & Teicher, 2004). The delayed effect of childhood adversity on hippocampal volumes might be due to hippocampal characteristics (Andersen & Teicher, 2004; Paquola et al., 2017) or, alternatively, related to the long‐term effects of childhood maltreatment on the biological stress response system (Andersen & Teicher, 2004; Murphy et al., 2022). Childhood maltreatment acts as a severe chronic stressor and is linked to long‐lasting alterations in the biological stress response system in afflicted individuals (e.g., Koss & Gunnar, 2018; McCrory et al., 2011; Murphy et al., 2022; Ouellet‐Morin et al., 2019). Prolonged stress exposure reduces neurogenesis in the hippocampus. Prolonged stress also stimulates hippocampal dendritic atrophy, loss of synapses and apoptosis of cells. Altogether, chronic stress exposure causes hippocampal volume reductions (Krugers et al., 2010; Sapolsky, 2000). A reduced hippocampal volume leads to prolonged elevated stress hormone exposure (“glucocorticoid cascade hypothesis”) (Frodl & O'Keane, 2013; Sapolsky, 2000) and renders the individual more susceptible to subsequent stressors over the life course (Gorka et al., 2014; Krugers et al., 2010; Weissman et al., 2020; Woon & Hedges, 2008): A vicious cycle of increasing stress susceptibility and decreasing hippocampal volumes can emerge whose effects accumulate over time and increase the risk of developing a psychiatric disorder.

Hippocampal volume alterations might become visible after adolescence because adolescence poses novel challenges to the individual: Puberty and young adulthood are periods of growing environmental demands and significant transitions (Cicchetti & Rogosch, 2002; Steinberg & Morris, 2001). Forming romantic relationships is an important developmental task at this age and a central aspect of young adults' lives (Furman & Shaffer, 2003; Gonzalez Avilés et al., 2021). At this young age, the stability of romantic relationships is often limited (Collins et al., 2009), and the breakup of a romantic relationship is rated as a very stressful life event (del Palacio‐González et al., 2017; Field, 2017). Childhood maltreatment puts adolescents at risk of experiencing more hardship in handling these developmental tasks (Herman, 1992). For instance, maltreated adolescents have difficulties in developing a positive and stable sense of self (Cederbaum et al., 2020; Kerber et al., 2023). Compared to non‐maltreated adolescents, they are more insecurely attached (Negriff et al., 2020), they receive less social support (McCrory et al., 2019; Negriff et al., 2020; Struck et al., 2020) and they have less stable interpersonal relationships (Kerber et al., 2023). In adulthood, maltreated individuals are more likely to experience a romantic relationship breakup/divorce compared to adults without childhood maltreatment (Whisman, 2006). In addition, emotional reactivity to stressful life events is higher in maltreated individuals, partly due to difficulties in emotion processing (e.g., higher levels of alexithymia) (Ditzer et al., 2023; Heleniak et al., 2016). Maltreated individuals engage in more maladaptive cognitive and behavioural coping responses such as rumination and impulsivity (Heleniak et al., 2016; Weissman et al., 2019). Accordingly, less resilience, more distress, more psychopathological symptoms (Francoeur et al., 2020) and higher levels of breakup‐related grief (Heshmati et al., 2021) were associated with the severity of childhood maltreatment after the experience of a romantic relationship breakup in young adults.

How a romantic relationship breakup might be related to hippocampal volumes in individuals with early life adversity is still unknown. It is conceivable that the stressful experience of a romantic relationship breakup impacts hippocampal volumes more strongly in maltreated compared to non‐maltreated individuals.

In this study, we hypothesized that a higher level of childhood maltreatment is associated with smaller hippocampal volumes in individuals that have experienced a romantic relationship breakup compared to those without a romantic relationship breakup. To this end, we investigated in 196 young healthy adults how childhood maltreatment interacts with romantic relationship breakup(s), experienced across the individual entire life span, on hippocampal volumes.

## 2 METHODS

### 2.1 Participants

Magnetic resonance imaging (MRI) structural data were collected from 198 healthy participants of a comprehensive research project (“Cultural Neuroscience”). This research project investigated psychological group processes with a cross‐sectional randomized experimental design. A detailed description of the experimental design is provided in a previous publication (Krautheim et al., 2019). The experimental manipulation as well as other collected data, such as genetic and fMRI data, are of no relevance in the presented study. Inclusion criteria of the research project were student status, age (18–40 years), right‐handedness (as assessed by the Edinburgh Inventory, Oldfield, 1971, inclusion criterion > + 40), German as a native language (to avoid heterogeneity in language processing in fMRI tasks) and Western‐ or Middle‐European descent (to increase homogeneity for genetic analyses). Exclusion criteria were history of major psychiatric disorders of participants and their first‐degree relatives according to ICD‐10 (assessed by the Mini‐International Neuropsychiatric Interview, Ackenheil et al., 1999), neurological or other relevant medical diseases (i.e., diseases that influence investigated parameters or jeopardize the participant's health during the investigation, such as coagulopathy) and metal implants or other MRI contraindications. Psychology students were excluded from participation in the project to avoid bias due to their possible knowledge of experimental fMRI task manipulation strategies. All participants were students at the Universities of Marburg or Gießen (Germany). All individual participants gave written informed consent, and the study protocol was approved by the local ethics committee of the Philipps University Marburg according to the Declaration of Helsinki (reference number N/KKS). Two participants were excluded from the analysis of structural data because of low data quality due to motion artefacts (see 2.2.4). The characteristics of the remaining 196 participants included in the analyses were as follows: 50% women; mean age = 24.0 years, SD = 3.2, range 19–38. A total of 20.6% were living with a partner (missings: n = 2).

### 2.2 Measures and procedure

All questionnaires were administered prior to scanning (in general at least one day beforehand).

#### 2.2.1 Relationship breakup

We assessed the occurrence and number of romantic relationship breakups and the separation from a spouse using two items from the List of Threatening Experiences Questionnaire (LTE‐Q, items 5 and 6) (Brugha & Cragg, 1990). No participant in our sample reported the separation from a spouse. Hence, the experience of a relationship breakup (in the following abbreviated with *breakup*) refers to the breakup of a steady committed romantic relationship (item: “Haben Sie jemals die Trennung einer eigenen festen Partnerschaft erlebt?”^1^). The *breakup* was assessed in yearly intervals. The number of experienced *breakups* was highly skewed to the right (range 0 to 4). We computed a dichotomous variable dividing the sample into participants with no *breakup* versus those with at least one *breakup* (*breakup* = 0: n = 98, versus *breakup*> 0: n = 98), occurring at any time till the time of investigation; the youngest reported age of a *breakup* was 15 years. To control for possible modulatory effects of the individual number of previous *breakups* (Benetti et al., 2010; Biondi & Picardi, 1996), we assessed the total number of *breakups* (n = 189, missings: n = 7). We also assessed the time interval since the last *breakup* (n = 92, missings: n = 6, range: 0–10 years).

#### 2.2.2 Childhood trauma

We assessed childhood maltreatment with the Childhood Trauma Screener (CTS) (Grabe et al., 2012). The CTS contains five items measuring emotional abuse, physical abuse, sexual abuse, emotional neglect and physical neglect. We summed up the scores of all CTS items to create a continuous sum score (CTS Sum). According to Witt et al. (2022), scores of >1 for the sexual abuse subscale and scores >2 for the other subscales are considered as a “warning signal” reflecting elevated clinical risk. We additionally created a dichotomized CTS risk score (0/1) that indicated if at least one of the five subscales scored higher than these cutoffs (CTS Risk = 1) or not (CTS Risk = 0). Further, we computed continuous scores for the experience of childhood threat and deprivation, by summing up the items of emotional, physical and sexual abuse (CTS Threat) and of emotional and physical neglect (CTS Deprivation) respectively.

#### 2.2.3 Control variables

We assessed clinical variables, personality traits and other stressful life events that were possible confounding factors in our study:

As clinical variables, we considered general anxiety and depressive symptoms. We administered the German versions of the State–Trait Anxiety Inventory, Trait version (STAI‐T;Laux et al., 1981; Spielberger et al., 1970) (Cronbach's α = 0.90) and the Beck Depression Inventory (BDI; Beck & Steer, 1987; Hautzinger et al., 1994) (Cronbach's α = 0.61).

We measured the personality trait alexithymia by using the German version of the Toronto Alexithymia Scale (TAS‐20) (Bach et al., 1996; Bagby, Parker, & Taylor, 1994; Bagby, Taylor, & Parker, 1994) (Cronbach's α = 0.81). Further, we administered the German version of the Relationship Scales Questionnaire (Griffin & Bartholomew, 1994; Stellmacher et al., n.d.). We analysed the individual adult attachment style according to the two‐dimensional model of adult attachment style proposed by Simpson (Simpson et al., 1992). This two‐dimensional model defines anxiety (ANX) and avoidance (AV) as two orthogonal axes (see also Kurdek, 2002). RSQ items were rated by the probands using a 6‐point scale and were reverse‐coded when necessary. We created the composite mean scores for the attachment dimension “avoidance” (AV) by averaging eight item scores and for “anxiety” (ANX) by averaging five item scores.

In a sociodemographic questionnaire, we evaluated whether participants were living with a partner (n = 194, missings: n = 2).

Finally, we determined other losses and stressful life experiences:

- The loss of a first‐degree relative or spouse (yes / no), and the loss of a close friend or close relative due to bereavement (yes/no) (List of Threatening Experiences Questionnaire; LTE‐Q, items 3 and 4) (Brugha & Cragg, 1990),
- The separation or divorce of parents up to the participant's age of 18 (yes/no).

#### 2.2.4 Acquisition of MR images

Data were acquired on a 3 Tesla whole‐body scanner (Siemens MAGNETOM Trio– A Tim System, Germany) at the Department of Psychiatry, University of Marburg. A three‐dimensional (3D) fast gradient echo sequence (GRAPPA) was used to acquire T1‐weighted high‐resolution anatomical images (repetition time = 1900 msec, echo time = 2.52 msec, flip angle = 9°, long‐term averages, inversion pre‐pulse every 900 msec, the field of view of 256 (feet‐head [FH]) × 256 (anterior–posterior [AP]) × 176 (right–left [RL]) mm, phase encoding in AP and RL direction, voxel size = 1 mm × 1 mm × 1 mm).

#### 2.2.5 MRI preprocessing and brain structure segmentation for the region‐of‐interest analyses

The native anatomical images were preprocessed and segmented by applying the volBrain pipeline (Manjón & Coupé, 2016). The pipeline includes the following steps: 1. Spatially adaptive non‐local means denoising, 2. rough inhomogeneity correction, 3. affine registration to MNI space, 4. fine SPM‐based inhomogeneity correction, 5. intensity normalization, 6. non‐local intracranial cavity extraction, 7. tissue classification, 8. non‐local hemisphere segmentation and 9. non‐local subcortical structure segmentation. The subcortical structure segmentation was performed applying non‐local label fusion (for details please refer to Coupé et al., 2011; Manjón & Coupé, 2016). Image processing quality and segmentation were visually assessed for all participants by using volBrain reports. Two participants were excluded because of low image quality: Images were ghosted and blurry due to motion artefacts. We used the uncorrected volumes of left and right hippocampi as well as total intracranial volumes (TIV) as provided by the volBrain segmentation in the statistical analyses (2.2.6).

#### 2.2.6 Statistical analyses

Statistical analyses of descriptive information and hippocampal volume data (2.2.5) were performed using R 4.2.0 (http://www.r-project.org/) (R Core Team, 2016). Packages in use were “Hmisc” (Harrell, 2022), “nortest” (Gross & Ligges, 2015), “ggplot2” (Wickham, 2016), “car” (Fox & Weisberg, 2019) and “psych” (Revelle, 2022) among others. The alpha level of statistical significance was set at *p* < 0.05 for all analyses. To control for the error rate related to multiple comparisons, we additionally report a false‐discovery‐rate (FDR) correction. We used the FDR correction for the two outcome measures (left/right hippocampi) (p.adjust function in R). We checked the assumptions of the multiple regression analyses by visual inspection of the correct specification of the model (Residuals vs Fitted plot, Lowess line), the normal distribution of the residuals (Normal Q‐Q plot), the homoscedasticity (Scale‐Location diagram) and critical outliers (Residuals vs. Leverage plot, Cook's distance) (‘plot’ function in R). Assumptions were met in all analyses. Homoscedasticity was additionally tested by means of the Goldfeld‐Quandt‐test; a p‐value > 0.05 indicates homoscedasticity.

##### 2.2.6.1 CTS sum scores, breakup and subcortical volumes

We performed standard multiple regression analyses to probe the association of CTS scores, *breakup* and bilateral hippocampal volumes. Four models were applied, for left and right hippocampal volumes respectively: 1. The basic model, containing the three control variables age, sex (women coded as 0, men as 1) and total intracranial volume (model B). These three control variables were included in every model. 2. The main effect of CTS Sum scores (model CTS). 3. An additive model of CTS Sum and the dichotomized *breakup* variable (*breakup* = 0 versus *breakup* > 0) (model “CTS + breakup”). 4. An interaction model of CTS Sum scores and the dichotomized *breakup* variable (*breakup* = 0 versus *breakup* > 0) (model “CTS x *breakup*”). An analysis of variance (ANOVA) model comparison was applied to determine whether each model added explanatory value over the reduced model (see Halldorsdottir et al., 2019): In the first comparison, model B was compared to model CTS, model CTS was compared to model “CTS + *breakup*” and model “CTS + *breakup*” was compared to model “CTS x *breakup*”. Models that significantly differed from the reduced model were further investigated in multiple regression analyses. We report the estimates, standard errors and p‐values of significant predictors.

We repeated the multiple regression analyses for significant results to control for clinical symptoms, attachment‐style, alexithymia, living with a partner and other loss experiences, by additionally including the following covariates in six independent control analyses: 1. depressive symptoms (BDI) and trait anxiety (STAI‐T), 2. adult attachment styles anxiety and avoidance, 3. alexithymia, 4. living with a partner, 5. parental separation and the loss of a first‐degree relative /spouse and/or close relative/close friend, 6. the total number of *breakups*. As three outliers (> ± 3 SD) of CTS Sum were observed we repeated the analyses without these three outliers in additional control analyses.

##### 2.2.6.2 Other CTS variables, breakup and subcortical volumes

In separate exploratory ANOVA model comparison analyses, we investigated the association of a) CTS Risk scores, b) CTS Threat and c) CTS Deprivation scores with hippocampal volumes as described in 2.2.6.1.

##### 2.2.6.3 Analyses of sex differences

Furthermore, we explored sex differences by analysing the interaction of a) CTS Sum, b) CTS Risk, c) CTS Threat and d) CTS Deprivation scores with sex on hippocampal volumes in multiple linear regression models including age and total intracranial volume as control variables. We performed control analyses for significant results as described in 2.2.6.1.

## 3 RESULTS

### 3.1 Descriptive information

Table 1 shows the descriptive information stratified by the experience of a relationship breakup (*breakup*). Participants who experienced a *breakup* reported significantly higher attachment‐related anxiety and significantly lower alexithymia. No other significant differences related to *breakup* were found. CTS variables were partly significantly correlated with age, sex, depressive symptoms, anxiety, attachment‐related avoidance, alexithymia and parental separation, but CTS variables were not significantly associated with the number of romantic relationship breakups (Table 2). Right hippocampal volumes were positively associated with attachment‐related anxiety (β = 0.04 ± 0.02, p = 0.043).

**TABLE 1:** Sample descriptives.

	Whole sample	*Breakup* = 0	*Breakup* > 0	
	n = 196	n = 98	n = 98	p
Total number of *breakups* (n = 189)	0.68 ± 0.85	0 ± 0	1.42 ± 0.67	<0.001
Years since last *breakup* (n = 92)	‐	‐	3.10 ± 2.62	‐
Right hippocampus [cm ^3^ ]	4.04 ± 0.37	4.05 ± 0.32	4.03 ± 0.41	0.793
Left hippocampus [cm ^3^ ]	3.99 ± 0.37	3.99 ± 0.35	4.00 ± 0.40	0.833
Total intracranial volume [cm ^3^ ]	1471.48 ± 132.99	1484.27 ± 130.09	1458.68 ± 135.28	0.179
CTS sum	6.57 ± 2.06	6.49 ± 1.74	6.64 ± 2.35	0.605
CTS Risk (no/yes)	150/46	79/19	71/27	0.178
CTS Threat	3.60 ± 1.36	3.45 ± 1.00	3.76 ± 1.64	0.116
CTS Deprivation	2.96 ± 1.15	3.04 ± 1.12	2.89 ± 1.17	0.352
Sex (m/f)	98/98	43/55	55/43	0.087
Age	24.0 ± 3.2	23.7 ± 3.2	24.4 ± 3.2	0.118
BDI	2.29 ± 2.31	2.13 ± 2.27	2.45 ± 2.35	0.339
STAI‐T	1.75 ± 0.40	1.75 ± 0.39	1.75 ± 0.41	0.943
RSQ ‐ ANX	2.15 ± 0.96	2.01 ± 0.84	2.29 ± 1.04	0.040
RSQ ‐ AV	3.16 ± 0.74	3.24 ± 0.75	3.09 ± 0.74	0.143
TAS	43.56 ± 9.21	45.08 ± 8.73	42.03 ± 9.47	0.020
Cohabiting with a partner (no/yes, n = 194)	154/40	78/19	76/21	0.723
Parental separation/divorce (before 18 years of age) (no/yes)	142/54	77/21	65/33	0.055
Loss of a spouse /first‐degree relative / close relative/close friend (no/yes)	83/113	42/56	41/57	0.885

Means, standard deviations (SD) or frequencies are listed for main and control variables, for the whole sample and for those with and without a relationship breakup (variable *breakup)* separately. In the right column p‐values of these subsample differences are shown.

**Abbr.:** ANX: Attachment anxiety, AV: Attachment avoidance, BDI: Beck Depression Inventory, CTS: Childhood Trauma Screener, RSQ: Relationship Scales Questionnaire, STAI‐T: State–Trait Anxiety Inventory, Trait version, TAS: Toronto Alexithymia Scale.

**TABLE 2:** Relations between variables.

	CTS Risk	CTS Threat	CTS Depriv	Sex	Age	BDI	STAI‐T	RSQ ANX	RSQ AV	TAS	CoP	Par Sep	Loss	N breakups
CTS Sum	−14.42 *** (t)	0.85 *** (r)	0.79 *** (r)	−0.10 (t)	0.23 ** (r)	0.15 * (r)	0.27 *** (r)	0.07 (r)	0.23 ** (r)	0.14 * (r)	0.23 (t)	−3.29 ** (t)	−0.91 (t)	0.05 (r)
CTS Risk	‐	−9.24 *** (t)	−11.54 *** (t)	0.11 (chi)	−1.70 (t)	−1.07 (t)	−3.06 ** (t)	−0.58 (t)	−2.03 * (t)	−1.66 (t)	0.29 (chi)	0.77 (chi)	0.26 (chi)	−1.63 (t)
CTS Threat		‐	0.35 *** (r)	2.12 * (t)	0.19 ** (r)	0.18 * (r)	0.21 ** (r)	0.05 (r)	0.16 * (r)	0.01 (r)	0.48 (t)	−3.83 *** (t)	−1.49 (t)	0.13 (r)
CTS Depriv			‐	−2.72 ** (t)	0.19 ** (r)	0.06 (r)	0.24 *** (r)	0.06 (r)	0.22 ** (r)	0.24 *** (r)	−0.10 (t)	−1.39 (t)	0.12 (t)	−0.06 (r)

Pearson Product Moment correlation coefficients (r), chi‐square (chi) or t‐test t‐values (t) are presented for the statistical relationships between the CTS variables and the control variables.

**Abbr:** CoP = Cohabiting with a partner; CTS Depriv = CTS Deprivation; ParSep = Parental separation/divorce before 18 years of age; Loss = Loss of a spouse /first‐degree relative / close relative/close friend, N breakups = number of relationship breakups.

*** : p < 0.001 (red).

** : p < 0.01 (orange).

* : p < 0.05 (yellow).

### 3.2 Hippocampal volumes, CTS Sum and *breakup*

**FIGURE 1:**
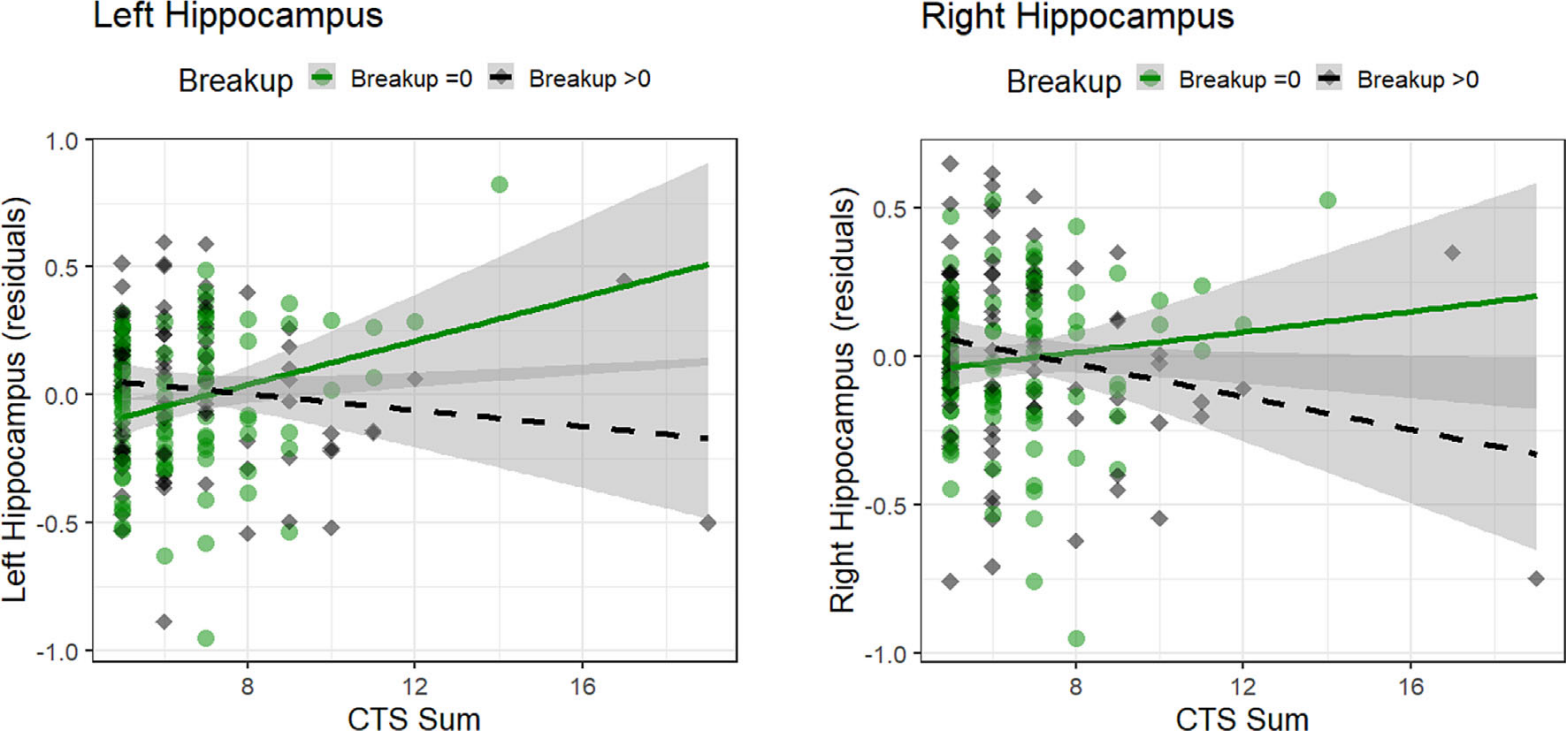
Interaction effect of CTS Sum and the experience of at least one relationship breakup (*breakup)* on hippocampal volumes. The associations of CTS Sum with grey matter volumes of bilateral hippocampi (residuals, controlling for age, sex and TIV) are depicted for *breakup* (*breakup* = 0/*breakup*>0). Significantly smaller bilateral hippocampal volumes were observed for higher CTS Sum scores in persons with *breakup* >0 compared to no *breakup* (N = 196; left hippocampus: β = −0.06 ± 0.02, p = 0.005, p (FDR) = 0.009; right hippocampus: β = −0.05 ± 0.02, p = 0.031, p (FDR) = 0.031).

The ANOVA model comparisons did not yield a significant main effect of CTS Sum on left or right hippocampal volumes, that is, model CTS did not add significant explanatory value (both p > 0.22) to the basic model. The same held true for the additive model “CTS + *breakup*”. By contrast, we observed that the model “CTS x breakup” significantly differed from the additive model “CTS + breakup” for both right and left hippocampal volumes, indicating significant CTS Sum‐by‐*breakup* interaction effects. The multiple regression models revealed that a higher CTS Sum score was associated with significantly smaller bilateral hippocampal volumes in participants with *breakup* compared to those with no *breakup* (Figure 1; Table 3; CTS Sum x *breakup*: right hippocampal volumes: β = − 0.05 ± 0.02, p = 0.031, p (FDR) = 0.031; left hippocampal volumes: β = −0.06 ± 0.02, p = 0.005, p (FDR) = 0.009).

**TABLE 3:** Interaction effect of childhood trauma and a relationship breakup (*breakup* = 0/*breakup* > 0) on hippocampal volumes.

	Left hippocampus [cm^3^]			Right hippocampus [cm^3^]		
	β ± SE	P (p_FDR_)	ES [%]	β ± SE	p (p_FDR_)	ES [%]
CTS Sum	−0.06 ± 0.02	0.005 (0.009)	41.6	−0.05 ± 0.02	0.031 (0.031)	41.2
CTS Risk	−0.24 ± 0.10	0.016 (0.018)	41.4	−0.23 ± 0.10	0.018 (0.018)	41.1
CTS Threat	−0.07 ± 0.04	0.034 (0.069)	40.4	−0.05 ± 0.03	0.187 (0.187)	40.6
CTS Depriv	−0.09 ± 0.04	0.019 (0.036)	41.1	−0.08 ± 0.04	0.036 (0.036)	40.8

The β‐estimates and standard errors (SE) of the multiple regression analyses, controlling for TIV, age and sex, are listed for the association of the Childhood Trauma Screener (CTS) variables with hippocampal grey matter volumes. The p‐value after FDR correction for multiple comparisons, correcting for the two subcortical outcome measures, is reported in parentheses. The adjusted effect sizes (ES) of the linear models are also displayed.

The interaction effect stayed significant in all control analyses and after correction for multiple comparisons for left hippocampal volumes, but was reduced to non‐significance for right hippocampal volumes after controlling for living with a partner (p = 0.156) and by the exclusion of the three CTS Sum outliers (p = 0.144).

We performed post hoc analyses for right and left hippocampal volumes respectively by splitting the sample into two groups (*breakup* = 0 vs. *breakup* > 0). Right hippocampal volumes were non‐significantly weakly positively correlated with CTS Sum scores in persons with no *breakup* (β = 0.01 ± 0.02, p = 0.546), but non‐significantly negatively correlated in those with at least one *breakup* (β = − 0.02 ± 0.01, p = 0.092). Larger left hippocampal volumes were associated with higher CTS Sum scores in persons with no *breakup (*β = 0.04 ± 0.02, p = 0.024). In participants with at least one *breakup*, higher CTS Sum scores were non‐significantly weakly associated with smaller left hippocampal volumes (β = −0.01 ± 0.01, p = 0.418).

In additional post hoc analyses for right hippocampal volumes, we analysed the interaction effect of CTS Sum‐ by‐ *breakup* in two different subsamples, i.e., participants who lived with a partner and those who did not. We found that in participants who did not live with a partner, a more negative interaction term (CTS Sum x *breakup*) was observed (β = − 0.04 ± 0.02, p = 0.145), than in those living with a partner (β = 0.05 ± 0.05, p = 0.395). Participants living with a partner were significantly older (p = 0.002), they showed significantly less attachment‐related avoidance (p < 0.001) and attachment‐related anxiety (p < 0.001) and the time interval since the last reported *breakup* was significantly longer (p = 0.001, M = 4.7 years vs. M = 2.6 years).

Of note, the time interval since the last reported *breakup* was not significantly related to left or right hippocampal volumes in the subsample of those with a breakup experience (linear time terms: p > 0.42; quadratic time terms: p > 0.21).

### 3.3 Hippocampal volumes, other CTS variables and *breakup*

The ANOVA model comparisons did not yield significant main effects of CTS Risk, CTS Threat or CTS Deprivation on left or right hippocampal volumes (all p > 0.13). We did not observe significant results for the additive model, either. However, we found that the interaction models significantly differed from the additive models for both right and left hippocampal volumes for all CTS variables, except for CTS Threat on right hippocampal volumes (Table 3). In more detail, we yielded the following results in the interaction analyses:

#### 3.3.1 CTS Risk x *breakup*

**FIGURE 2:**
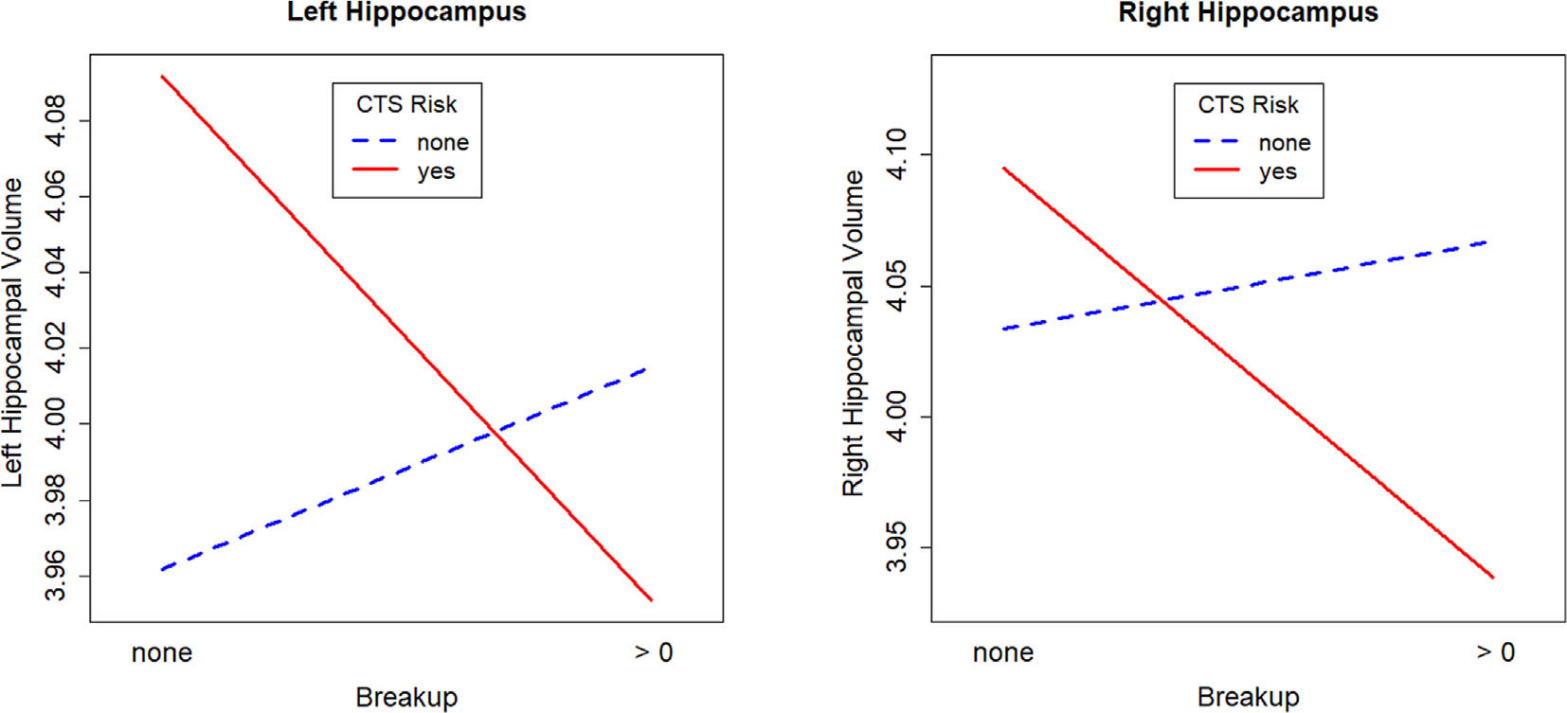
Interaction plot of CTS Risk and the experience of at least one romantic relationship breakup (*breakup)* on hippocampal volumes. Disordinal interactions between CTS Risk and *breakup* (*breakup* = 0/*breakup*>0) were observed for both left and right hippocampal volumes [cm^3^] (N = 196; left hippocampus: β = −0.24 ± 0.10, p = 0.016, p (FDR) = 0.018; right hippocampus: β = −0.23 ± 0.10, p = 0.018, p (FDR) = 0.018).

With regard to CTS Risk, we observed significant disordinal interactions that survived FDR correction for multiple comparisons and all control analyses (Table 3, Figure 2). In the group with a given CTS Risk (CTS Risk = 1), left and right hippocampal volumes were smaller after at least one *breakup* compared to no *breakup*. The opposite pattern was found in the group with no CTS Risk: Hippocampal volumes were larger, even though less pronounced, in association with a *breakup* compared to no *breakup*.

#### 3.3.2 CTS Threat x *breakup*

A higher CTS Threat score was linked to significantly smaller left hippocampal volumes in persons with at least one *breakup* compared to those without (Table 3).

The interaction effect did not survive the correction for multiple comparisons. In the control analyses, the CTS Threat‐by‐*breakup* interaction effect was non‐significant for left hippocampal volumes after exclusion of CTS Sum outliers (p = 0.338) and after controlling for living with a partner (p = 0.171; post hoc: CTS Threat x *breakup*: Living without a partner: β = − 0.06 ± 0.04, p = 0.213, living with a partner: β = − 0.06 ± 0.07, p = 0.407).

#### 3.3.3 CTS Deprivation x *breakup*

A higher CTS Deprivation score was related to significantly smaller left and right hippocampal volumes in persons with at least one *breakup* compared to those without *breakup*.

Both interaction effects survived the correction for multiple comparisons. In the control analyses, the CTS Deprivation‐by‐*breakup* interaction effect was non‐significant for right hippocampal volumes after exclusion of CTS Sum outliers (p = 0.063) and after controlling for living with a partner (p = 0.069; post hoc: CTS Deprivation x *breakup*: Living without a partner: β = − 0.09 ± 0.04, p = 0.030, living with the partner: β = 0.06 ± 0.07, p = 0.415).

### 3.4 Exploration of sex differences

We did not detect significant sexually dimorphic interaction effects on hippocampal volumes, neither for CTS Sum (both p > 0.44), nor for CTS Risk, CTS Threat or CTS Deprivation (all p > 0.15).

## 4 DISCUSSION

Childhood trauma is a known vulnerability factor for the development of psychiatric disorders (Bailey et al., 2018; McGinnis et al., 2022), but the pathway from childhood trauma to mental illness is not yet fully understood. We hypothesized that childhood maltreatment increases the individual's vulnerability to a stressor in adolescence/young adulthood. We further hypothesized that the interaction between childhood maltreatment and stress might be associated with hippocampal volume alterations that resemble those observed in psychiatric disorders. To shed more light on this putative vulnerability‐stress‐interaction, we investigated the association between retrospectively reported childhood maltreatment, the experience of a breakup of a steady committed romantic relationship (*breakup*) and hippocampal grey matter volumes in young adults.

Our results showed that childhood maltreatment per se was not associated with hippocampal volumes in young adults. However, we found a dose–response relationship between higher childhood trauma scores (CTS Sum) and smaller bilateral hippocampal volumes in participants who experienced at least one *breakup* compared to those with none. Participants who did not report a *breakup* displayed a positive association between the severity of childhood maltreatment and left hippocampal volumes.

We also compared maltreated to non‐maltreated individuals by using a dichotomized CTS risk score according to reported cut‐off values of CTS subscales (Witt et al., 2022). We found that maltreated participants exhibited smaller bilateral hippocampal volumes after at least one *breakup* compared to no *breakup*. The opposite pattern was observed in non‐maltreated participants.

In all our analyses, interaction effects were slightly stronger for left compared to right hippocampal volumes. Interaction effects were also slightly stronger for childhood deprivation compared to childhood threat experiences. Furthermore, adults who were living with their romantic partner at the time of investigation exhibited partly weaker interaction effects between CTS variables and *breakup* on hippocampal volumes.

In sum, our results support our hypothesis that stressful experiences in adolescence/young adulthood moderate the association between childhood maltreatment and hippocampal volumes in young adults. As hypothesized, we found a dose–response‐relationship between the severity of childhood maltreatment and hippocampal volume reductions in persons with *breakup*. In persons with *breakup*, w*e* also observed a categorical relationship between childhood maltreatment and smaller hippocampal volumes, providing further support to our results.

Reduced hippocampal volumes, especially of the left hemisphere, present a common feature in many psychiatric conditions such as MDD, schizophrenia and bipolar disorders (Brosch et al., 2022). It has been put forward that hippocampal volume reductions mediate the effects of long‐term stress on mental health risk (Frodl & O'Keane, 2013; Opel et al., 2014).

The results of our morphometric interaction analyses align with the findings of behavioural studies of romantic relationship breakups (Francoeur et al., 2020; Heshmati et al., 2021). These studies showed dose–response relationships between the level of childhood maltreatment and the severity of breakup‐related grief, distress and psychiatric symptoms in young adults.

We did not observe main effects of childhood maltreatment on hippocampal volumes. The young mean age of our sample places our study between studies of adolescents and adults: studies in adolescents yielded inconsistent associations between childhood maltreatment and hippocampal volumes (Lee et al., 2018; Malhi et al., 2019; Whittle et al., 2013, 2017). Studies in middle‐aged adults including meta‐analyses consistently linked childhood maltreatment to adult hippocampal volume reductions, either by group comparisons (Paquola et al., 2016) or in associative studies (Opel et al., 2014; Riem et al., 2015). Thereby, we suggest that in the transitional period between adolescence and middle‐aged adulthood, the experience of childhood maltreatment itself is not linked to hippocampal structural alterations; however, hippocampal volume reductions become evident after the occurrence of additional stressors, such as difficulties in mastering normative developmental tasks (Francoeur et al., 2020; Heshmati et al., 2021).

Interestingly, a recent behavioural study in a large sample of middle‐aged adults (n = 2363) (Kerber et al., 2023) showed that the association between adverse childhood experiences and psychopathology was moderated by personality functioning, i.e., the stability of the self and of interpersonal relationships: Low levels of personality functioning rendered maltreated individuals more vulnerable to psychopathology, while high levels of personality functioning conveyed more resilience in the face of adversity (Kerber et al., 2023). We postulate that the occurrence of a relationship breakup is partly related to the level of personality functioning in maltreated individuals. We propose that a higher level of personality functioning in our group of maltreated individuals without *breakup* compared to those with *breakup* renders them more resilient to psychopathology. This might underlie our surprising observation that more severe childhood trauma was associated with larger left hippocampal volumes in the group without *breakup*. In studies of adults, larger hippocampal volumes were associated with more resilience against PTSD and depression (Chan et al., 2016; Rubin et al., 2016).

Furthermore, our data suggest that the experience of a stable romantic relationship partly acts as a resilience factor in the face of childhood maltreatment and past relationship breakups. In our study, the experience of a stable committed romantic relationship at the time of investigation mitigated the association between childhood maltreatment, *breakup* and hippocampal volumes. Participants who were living together with their partner at the time of investigation also reported lower levels of attachment‐related anxiety and avoidance. The attachment styles AV and ANX are known to increase vulnerability to depressive symptoms in maltreated individuals (Struck et al., 2020). Attachment insecurity compromises resilience, partly mediated by higher emotion suppression (Fritz et al., 2018; Heshmati et al., 2021). However, our results did not change after controlling for the adult attachment styles AV and ANX. Hence, we postulate that the experience of a stable romantic relationship rather than adult attachment styles affects the association between the level of childhood maltreatment, *breakup* and hippocampal volumes. The relevance of social affiliations in the face of adversity was demonstrated in recent primate studies. These studies revealed that strong social bonds can buffer long‐term negative effects of early adversity (Lange et al., 2022; Morrison et al., 2023).

In the past decade, there has been an onging debate on the dimensionality of childhood maltreatment (Brieant et al., 2022). McLaughlin et al. (2014) proposed to distinguish between childhood deprivation and threat experiences. Deprivation and threat often co‐occur in families but can be measured independently (McLaughlin et al., 2014). According to their theory threat experiences, but not deprivation experiences were expected to affect hippocampal structures. However, our study results did not confirm this hypothesis, showing similar and even partly stronger interaction effects on hippocampal volumes for CTS Deprivation than CTS Threat.

Our study highlights the clinical importance of stable social bonds for individuals with childhood maltreatment experiences. Maltreated individuals might profit from clinical interventions such as psychotherapy that help them establish stable relationships and a stable sense of self. Our study provides some evidence that stable relationships in young adulthood can mitigate sequelae of early life adversity and might increase resilience to psychopathology.

### 4.1 Limitations

Childhood trauma and the number of relationship breakups were assessed retrospectively. Prospective and retrospective measures of childhood maltreatment only partially converge (Baldwin et al., 2019; Reuben et al., 2016). The discrepancy between prospective and retrospective assessments is presumably based on methodological issues, individual memory formation and motivation of disclosure (Coleman et al., 2024). While both prospective and retrospective assessments predict psychopathology, the strongest associations were found with retrospective instruments (Reuben et al., 2016). Both overreporting (false positives) and underreporting (false negatives) are possible in retrospective assessments (Coleman et al., 2024), and it has been put forward that underreporting is more likely than overreporting (Hardt & Rutter, 2004). Compared to the Childhood Trauma Questionnaire (Bernstein et al., 2003) the CTS does not contain validity items to detect the minimization or denial of trauma. Hence, we cannot rule out that underreporting of childhood maltreatment biased the results of our study. Furthermore, the CTS is a short screening instrument which limits its reliability compared to longer versions of childhood trauma questionnaires. The CTS also does not assess the timing or chronicity of trauma exposure which both have an impact on the maltreatment‐related susceptibility of the hippocampus (Teicher et al., 2016). However, the CTS highly correlates with longer questionnaires such as the Adverse Childhood Experiences Questionnaire and the Childhood Trauma Questionnaire (Grabe et al., 2012; Witt et al., 2022). The CTS is considered a valid, reliable and economic instrument (Grabe et al., 2012; Witt et al., 2022). Given the comprehensive data collection in our research project, we used the CTS because of its time efficiency.

We investigated a representative sample of students. We included only students in our study to achieve a homogeneous sample: while this strategy reduces potential confounder effects, it also limits the generalizability of our study results. Given the high education and absence of mental disorders in our sample, it is likely that highly resilient individuals were overrepresented in the group of maltreated participants.

Our analyses captured around 40% of the variance in hippocampal volumes. Hippocampal volumes are also shaped by other factors such as genetic variants which we did not include in our analyses (Janowitz et al., 2014). We only investigated hippocampal volumes. However, structures of other brain regions such as cingulate and striatum are altered as well by both childhood maltreatment and psychopathology (e.g., Price et al., 2021; Teicher et al., 2016; Zhang et al., 2018).

It would be worthwhile to replicate and extend our results in a comprehensive longitudinal study. This would allow to complement retrospective assessments of childhood trauma and romantic relationship experiences by prospective measures. For the retrospective assessment of childhood trauma, more detailed measures would be desirable that also assess the timing and chronicity of childhood maltreatment. Furthermore, future work that analyses more brain regions could inform us about whether our findings are unique to the human hippocampus. Future studies that consider genetic data could reveal gene–environment interactions between adversity and hippocampal volumes. Finally, it would be interesting to address our research questions in the general population and/or patient populations to provide more universal and transferable results.

### 4.2 Conclusions

Hippocampal volumes might represent a connecting structure between childhood maltreatment and adult psychopathology. Our study in young adults showed that romantic relationship breakup experiences moderate the association between the level of maltreatment and hippocampal volumes. While our study was cross‐sectional and no causal relationships can be inferred, our results conform with the hypothesis that childhood maltreatment renders an individual more sensitive to subsequent stressors in life such as coping with normative developmental tasks. Moreover, our study highlights the relevance of social affiliations in adulthood in the face of early adversity, translating research from primates to humans. Our findings further underline the relevance of therapeutic interventions for maltreated individuals that address the individual's capacity to cope with loss and to establish and maintain strong social bonds.

## AUTHOR CONTRIBUTIONS

**HA**: Conceptualization; data curation; formal analysis; investigation; methodology; project administration; visualization; writing—original draft. **AJ**: Data curation; resources; software; supervision; writing—review and editing. **TK**: Funding Acquisition; project administration; resources; supervision; writing—review and editing.

## CONFLICT OF INTEREST STATEMENT

The authors declare that they have no conflict of interest.

### PEER REVIEW

The peer review history for this article is available at https://www.webofscience.com/api/gateway/wos/peer-review/10.1111/ejn.16593.

## ETHICS STATEMENT

All procedures performed in studies involving human participants were in accordance with the ethical standards of the institutional and/or national research committee and with the 1964 Helsinki declaration and its later amendments or comparable ethical standards.

## INFORMED CONSENT

Informed consent was obtained from all individual participants included in the study.

## Data Availability

The data that support the findings of this study are available from the corresponding author, HA, upon reasonable request. The data are not publicly available due to ethical restrictions. Informed consent of the study did not include sharing of the data in a public repository. Compliance with Ethical Standards:

## Footnotes

^1^
Item translation: “Have you ever experienced the breakup of a steady committed romantic relationship?”. The German term” feste Partnerschaft” is an idiomatic expression for a steady committed romantic relationship.
